# Tailoring Science Outreach through E-Matching Using a Community-Based Participatory Approach

**DOI:** 10.1371/journal.pbio.1001026

**Published:** 2011-03-08

**Authors:** Bernice B. Rumala, Jack Hidary, Linda Ewool, Christopher Emdin, Ted Scovell

**Affiliations:** 1 The Rockefeller University, New York, New York, United States of America; 2 Columbia University, Teachers College, Department of Mathematics, Science and Technology, New York, New York, United States of America; 3 National Lab Network Organization, New York, New York, United States of America; 4 Renaissance High School, Ecology and Chemistry Teacher, Bronx, New York, United States of America

**Summary**

In an effort to increase science exposure for pre-college (K–12) students and as part of the science education reform agenda, many biomedical research institutions have established university–community partnerships. Typically, these science outreach programs consist of pre-structured, generic exposure for students, with little community engagement. However, the use of a medium that is accessible to both teachers and scientists, electronic web-based matchmaking (E-matching) provides an opportunity for tailored outreach utilizing a community-based participatory approach (CBPA), which involves all stakeholders in the planning and implementation of the science outreach based on the interests of teachers/students and scientists. E-matching is a timely and urgent endeavor that provides a rapid connection for science engagement between teachers/students and experts in an effort to fill the science outreach gap. National Lab Network (formerly National Lab Day), an ongoing initiative to increase science equity and literacy, provides a model for engaging the public in science via an E-matching and hands-on learning approach. We argue that science outreach should be a dynamic endeavor that changes according to the needs of a target school. We will describe a case study of a tailored science outreach activity in which a public school that serves mostly under-represented minority students from disadvantaged backgrounds were E-matched with a university, and subsequently became equal partners in the development of the science outreach plan. In addition, we will show how global science outreach endeavors may utilize a CBPA, like E-matching, to support a pipeline to science among under-represented minority students and students from disadvantaged backgrounds. By merging the CBPA concept with a practical case example, we hope to inform science outreach practices via the lens of a tailored E-matching approach.

*…Someday I want to be a scientist in the medical field and be successful in life. For that to become a reality I will have to take several steps in life, I know some of them but not all so I would love for you to become my mentor and guide me through some of the necessary steps for my dreams to become my life in the future. Like you I don’t come from the best neighborhood, so not many people around are the best role models for me to look up to. I’m not really sure which medical field just yet I'm still trying to decide but I just know that I want to make people feel better and possibly save lives. I really want to be in the science field because you don’t really see many Latino or Latina doctors walking around … I want you to know that at my school they don't give the science classes that I'm suppose to have by this time in my 10th grade year, this is very disappointing and scares me because I'll be behind everyone else in my later years. At times I wonder if I had the science classes that other students are getting if I would be interested in becoming part of the science field as I am now.*

— Message from a student who attends high school in Brooklyn, New York

## Introduction

Studies in science education indicate that exposure to science and science in practice may increase students’ interest in the discipline [1],[2]. Consequently, pre-college science outreach has grown to become an integral component of creating a generation of students that are scientifically literate and interested in pursuing science careers [3],[4]. As reliance on the efforts of pre-college programs has increased, and such efforts to increase visibility and outreach of these programs have been expanded, the need to ensure that these programs meet the goals they have intended by targeting the specific concerns of the school community is more urgent than ever [5]. To address this need, we suggest that E-matching is a useful tool to ensure mutually beneficial partnerships between students and scientists. E-matching, a term coined in this paper, is electronic web-based matching of teachers and their students with science, technology, engineering, and mathematics (STEM) experts. E-matching can occur based on interest, need, and/or geography. The E-matching approach focuses on the needs of students both within and outside of the classroom, has the goal of increasing students' participation in science, and specifically caters to pre-college science outreach needs. Since science is a discipline that consists of multiple entry points and various subdisciplines, E-matching allows students to explore their specific interests by gaining access to mentors and experts within these specific domains. In addition, it helps harness their scientific interests by providing biomedical expertise that will be beneficial throughout their academic careers.

## Tailoring Science Outreach: Community-Based Participatory Approach

E-matching embraces the community-based participatory approach (CBPA), which has its roots in the social sciences, public health, and education [6],[7]. Most recently, the CBPA has become a major focus of the Clinical Translational Science Award community engagement initiative [8]. The traditional science outreach programs typically involve a pre-structured science experience with little input from the teachers and students in the planning and implementation of the science outreach experience. However, applying the CBPA to science outreach entails a collaborative approach that engages all partners and stakeholders equally. In this project, stakeholders include high school science teachers, scientists, and science outreach administrators. Unlike the traditional approach, the CBPA acknowledges that each stakeholder has unique needs and contributes a unique strength to mutually benefit the partnership (see Table 1). The school benefits from a tailored science outreach experience and the scientists benefit from the stimulation of sharing research with inquisitive young minds and fulfilling broader impact objectives often required of grant submissions.

**Table 1:** Traditional approach versus CBPA through science outreach E-matching.

Traditional	CBPA
University^a^ has pre-structured outreach that may be formulated in consultation with members of the university.	Outreach is tailored and formulated in collaboration with representatives of the K–12 school to ensure that the needs of the student population are met. The university and the K–12 school are equal partners in the development of the science outreach plan.The K–12 school approaches the university with a science outreach need, or the university can approach the K–12 school with a science outreach proposal that can be tailored to the K–12 school's needs.
Impacts of science outreach are shared with members of the university.	Impacts of science outreach are shared with both members of the university and representatives of the K–12 school as part of collaborative partnership.
Sometimes relationship with the K–12 school ends after the outreach, especially if it is of short duration.	Relationship with the K–12 school continues.
Debriefing often occurs with members of the university.	Debriefing often occurs with the K–12 school and university as part of the collaborative endeavor.
Evaluation is done by the university on the K–12 school and shared with members of the institution.	Evaluation is done by the university in collaboration with the K–12 school. The results are shared with both the K–12 school and members of the university.
Less time is required to set up the program.	More time is required to set up program as a result of tailored approach.

a Note: For the purposes of this paper, university is used as the collaborating organization. However, the collaborating organization can extend beyond the university.

This CBPA begins by assessing the primary interest of the school. E-matching allows a school to advertise its needs so that outside institutions that can provide resources may contact the schools and subsequently tailor an agenda to fulfill those needs. This approach has many components that are designed to lead to more successful science outreach outcomes, as it (1) empowers the teacher with tools and resources to shape the science exposure needs of his or her students; (2) puts the needs of the school first; and (3) facilitates a dynamic endeavor since each relationship is tailored to the needs of the school and the resources of the scientist. Table 1 describes the process of tailoring science outreach endeavors utilizing a CBPA via E-matching and compares it to the traditional approach to science outreach.

## E-Matching with National Lab Network

The National Lab Network (NLN) initiative, formerly National Lab Day, is a worthwhile model for outreach initiatives that employs an E-learning approach. Recognizing a critical need to increase science equity and literacy, and employing a CBPA, NLN was created in 2009 as a national call to action. NLN tailors its outreach to the needs of the target student population via an E-matching service that links K–12 teachers with STEM professionals, organizations, and resources. However, NLN is more than just a day; it is an ongoing effort to increase the number of K–12 schools receiving outreach in the STEM disciplines.

**Figure 1:**
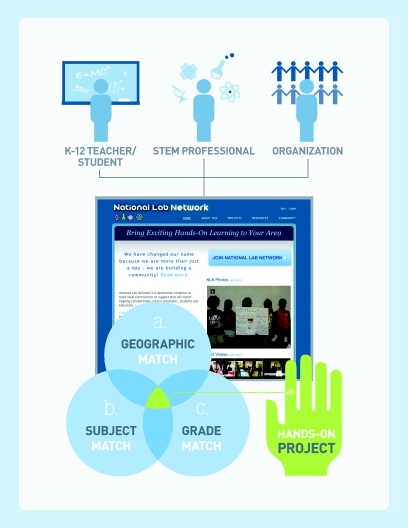
National Lab Network E-matching diagram.

**Figure 2:**
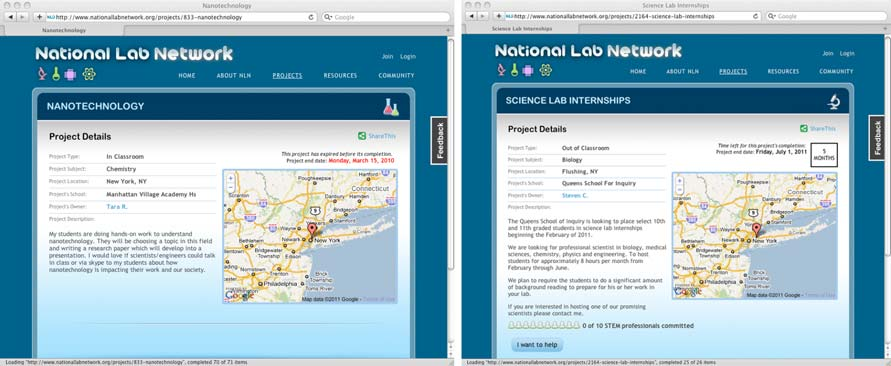
Ads from National Lab Network site.

The E-matching process occurs on the NLN website (http://www.nationallabnetwork.org) that links projects proposed by teachers with scientists and their resources, thereby building local communities of support. Scientists and teachers are matched on a variety of parameters including subject matter, geographic location, grade level, interests, need, and other factors (Figure 1). This model allows for teachers to connect with STEM experts who can tailor an outreach based on the specific needs of teachers and students. In addition to this linking process, all projects and registered users on the NLN website are accessible beyond suggested matches. This process serves as a model that allows for a CBPA that complements a traditional one. Figure 2 shows archived ads from teachers on the NLN website.

Websites have been used as a powerful tool for organizations to provide science outreach to the public by providing online educational resources [9], promoting hands-on learning [10] and virtual learning [11], and building a community of scientists [12]. The NLN model also complements existing efforts to create an online partnership between scientists and the public. Most of the web-based resources complement each other by fulfilling different science outreach needs. Two examples of such efforts are Ask a Biologist (http://www.askabiologist.asu.edu) [13] and MySciNet (http://community.sciencecareers.org/myscinet). Ask a Biologist is a forum sponsored by Arizona State University where students can ask scientists questions about biology. This site also includes virtual experiments. MySciNet allows scientists and students to discuss their general scientific interests and science careers. NLN enhances existing science web community efforts like these by directly linking students and teachers with experts who can provide both an E-learning experience and in-person, hands-on experiences within the classroom or at a collaborating institution. This hands-on learning provides additional options for teachers and students to receive authentic scientific training in the real world.

Employing the web for scientist–teacher–student matching has several advantages. First, the web platform provides a variety of resources that traditional matching services cannot deliver. Scientists who represent institutions can choose student projects or schools that they want to work with. E-matching also empowers the teacher/student to communicate their science interests through a platform that enables them to solicit and access resources and subsequently collaborate with one or more STEM experts. Second, the website provides personalization. Each teacher and scientist on the site has a personal dashboard and regular personal email updates that are customized to their goals and attributes. Third, teachers can either complement or fill a gap in their existing science curriculum with real world experiences from STEM experts. Fourth, the website allows for community engagement via rapid connections between the K–12 school and the STEM professional. Finally, using the web provides data for measurement and assessment that can inform science outreach program practices (Figure 1).

## Collaboration: An E-Matching Case Study

Through the E-matching platform provided by NLN, Rockefeller University collaborated with a New York City public high school in the Bronx. This Bronx high school's student body was largely made up of students who are of under-represented minority and from socioeconomically disadvantaged backgrounds. A teacher (L.E.) submitted a profile on the NLN website that indicated three major objectives: (1) An exposure to racially/ethnically and gender diverse scientists, (2) exposure to diverse career options in the sciences, and (3) an opportunity to see “real-life science” at work as a complement to the existing science curriculum.

The Rockefeller University Community Engagement Specialist accessed the NLN site and chose the school based on the following broader Rockefeller University National Lab Network objectives: (1) To provide science outreach to schools serving predominantly under-represented minority and/or disadvantaged students, (2) to tailor each outreach according to the specific needs of the school; (3) to serve as an information pipeline to inform students of research opportunities at The Rockefeller University and other institutions, (4) to connect students with mentors, (5) to expose students to diverse scientists/trainees with an emphasis on under-represented minority scientists/trainees, (6) to emphasize the importance of science and science literacy regardless of a student's career trajectory. After discussions with the stakeholders, a tailored agenda was formulated addressing the K–12 school areas of interest (Table 2).

**Table 2:** Science outreach needs and tailored agenda for National Lab Day.

School Needs	National Lab Day Agenda	Participants
Need for exposure to racially and ethnically diverse scientists	Mentoring advice; diverse science training and careers panel consisting of racial/ethnic and gender diverse scientists/scientists in training	PhD, MD/PhD, and MD students
Exposure to diverse career options in the sciences	Mentoring advice, science training and careers panel, mentoring and mingling session, campus/lab tours, interactive science demonstration, and science research presentation	Science outreach staff, research scientists, and various members of the health professional and research team
Seeing “real-life science” at work	Science research presentation, interactive science demonstration, lab tours	Science outreach staff; health professional and research team; PhD, MD/PhD, and MD students

In initiating the collaboration through a CBPA, both the K–12 school and the institution were bringing unique strengths to the endeavor. This approach also met the needs of all stakeholders in the partnership.

### K–12 Teacher's Perspective (L.E. – Ecology and Chemistry high school teacher): Science Outreach to Urban Youth via E-Matching

*The chances of my urban high school students interacting with science professionals increased dramatically with E-matching. The ability to propose a project on the internet, which entailed the interaction of science professionals with urban youth, allowed for the publication of the needs of my chemistry and ecology class. In addition, the logistics of physical location, grade level appropriateness and diversity which were apparent within the proposal allowed for feasible responses. Communication is a key component in education and the ability to electronically connect with professionals actively engaged within the content area of study has important ramifications.*

Students must work in a rich, responsive environment and have contact with knowledgeable people in the community if they are to learn important critical thinking skills [14].

*Several institutions responded to my proposal, and engaged my students in ways that allowed for collaborations both within and outside the school setting* [Table 2]. *As part of the outreach within the school setting, students targeted in my proposal listened to a presentation and interacted with a chemist from a translational research laboratory. As part of the out of school experience, students visited a biomedical research university (The Rockefeller University). Via personal testimonial, research presentation, science content demonstrations, interactive panel discussions, campus tours and meet and greet with a variety of researchers, students and professionals, students experienced the possibilities, activities and interactions which occur within a science educational research organization. Their knowledge base of the opportunities inherent within the scientific field and the connections between their understanding that what is learned in the classroom as an initiation to higher education settings and real world application, broadened immensely.*

*The benefits and opportunities which resulted from the relationships formed during this E-matching process (including acceptance of four students to the longer duration summer program) enhanced the awareness of my Bronx high school students to the scientific community. These connections were made possible due to the establishment of a venue in which partnerships could be initiated electronically. The fact that there were individual STEM professionals and their respective scientific organizations whom were willing and able to connect with urban youth and offer these students a window into the STEM community was made possible through the E-matching initiative. As the teacher I also received stimulation from these opportunities to connect with scientific and educational professionals engaged in the process of academic and medical research. Such interactions will serve to enhance the educational experiences of the students, teachers and the scientific community.*

## Preliminary Benefits

Outreach via E-matching has shown several benefits. It has served as an information pipeline for under-represented minority and disadvantaged students, enabling them to consider science careers as a viable option. As a result of the E-matching exposure, 19% (7) of the 36 student participants who previously had no exposure to science or real-world scientists went on to apply to The Rockefeller University Summer Neuroscience Program, and 57% (4) were accepted. Through establishment of an E-mentoring network the relationship has continued with the students who are interested in further discussions about science careers; this electronic mentoring network consists of Rockefeller University scientists, health professionals, staff, and research team members who have made their electronic contacts available for students to receive further guidance. Lastly, under the auspices of NLN, this collaborative effort has allowed for both institutional buy-in via funding for the initiative and community buy-in via navigating administrative logistics for a successful academic–community partnership. As a result of the community-based participatory E-matching approach, both the research institution and lay community had an equal investment in the outcome, because they are stakeholders at the beginning and are actively involved in formulating the agenda. This buy-in helps to encourage long-term sustainability of the outreach.

## Conclusion

Utilizing E-matching and a CBPA to tailor science outreach allows for highly specific, targeted, and collaborative coordination of science outreach endeavors to meet the needs of all stakeholders. Reflecting back on the high school student's statement at the beginning of this manuscript about inequities in science access, E-matching can address some of the science exposure disparities seen in many urban classroom settings. This approach may also serve as a pipeline to increase diversity in the STEM fields. We don't deny that for some science outreach programs a traditional, pre-structured outreach may work better. Perhaps a hybrid approach to science outreach utilizing both the traditional and tailored approaches would best meet the science outreach needs of our youth.

Considering internet access limitations in some communities, it is important to note that the tailored approach is not limited to E-matching but can also be done by connecting with under-resourced schools through other communications media to collaboratively plan a science outreach program. Both the institution and the school must be proactive in following up on the logistics for a successful collaboration. The CBPA tailored science outreach approach via the E-matching model may increase students' enthusiasm for science, serve as a pipeline for student participation in more science programs, and potentially increase student entry into STEM careers.

## Abbreviations

CBPA: community-based participatory approach
NLN: National Lab Network
STEM: science, technology, engineering, and mathematics
